# Porcine ear necrosis is associated with social behaviours in weaned piglets

**DOI:** 10.1186/s12917-024-03974-4

**Published:** 2024-03-23

**Authors:** Gwenaël Boulbria, Théo Nicolazo, Charlotte Teixeira-Costa, Caroline Clouard, Arnaud Lebret, Valérie Normand, Céline Chevance, Justine Jeusselin, Élodie Merlot

**Affiliations:** 1 REZOOLUTION Pig Consulting Services, Parc d’Activités de Gohélève, Rue Joseph et Étienne Montgolfier, Noyal-Pontivy, 56920 France; 2 PORC.SPECTIVE Swine Vet Practice, Parc d’Activités de Gohélève, Rue Joseph et Étienne Montgolfier, Noyal-Pontivy, 56920 France; 3 PEGASE, INRAE, Institut Agro, Le Clos, Saint Gilles, 35590 France

**Keywords:** Porcine ear necrosis, Ear lesion, Behaviour, Scan sampling, Oxidative stress, Biomarkers

## Abstract

**Background:**

Porcine ear necrosis (PEN) is a worldwide health issue and its aetiology is still unclear. The aim of this study was to describe the prevalence and the severity of PEN in a commercial farm, associated with pig behaviour and health biomarkers measures.

On two consecutive batches, PEN prevalence was determined at the pen level. PEN scores, blood haptoglobin concentration and oxidative status were measured on two pigs per pen (*n* = 48 pens) 9, 30 and 50 days (D) after arrival to the post-weaning unit. Social nosing, oral manipulation and aggression of pen mates and exploration of enrichment materials were observed on two to three pigs per pen twice a week from D9 to D50.

**Results:**

At the pen level, the higher the time spent nosing pen mates, the lower the percentage of pigs affected by PEN during both the early and the late post-weaning periods (*P* < 0.002) and, in the opposite, the higher the time spent orally manipulating pen mates during the late post-weaning period, the higher the percentage of affected pigs (*P* = 0.03). At the pig level, the higher the increase in hydroperoxides and haptoglobin during the early post-weaning period, the higher the PEN scores on D30 (*P* < 0.001).

**Conclusions:**

This study suggests that a high incidence of social nosing, which can be an indicator of good social cohesion in a group, was significantly associated with less frequent lesions of PEN. In opposite, high incidence of oral manipulation of pen mates may increase the percentage of PEN-affected pigs. According to these observations, PEN is a multifactorial condition which may have social causes among others.

## Background

Porcine ear necrosis (PEN) is an increasing health issue worldwide and a daily concern for farmers and swine veterinarian practitioners [1–5]. Moreover, it causes a major welfare issue for pigs. PEN is characterized by necrotizing ulcerative lesions on the tip of the pinna. Bleeding may also be present. Sometimes, it can result in the loss of part of or the entire ear. Under some circumstances, the percentage of pigs with severe lesions can be high, with studies reporting a prevalence of 30% of finisher pigs in Denmark [5] and 11–46% at the end of the nursery period in Belgium [6, 7].

However, PEN pathogenesis and associated risk factors are still little explored and poorly understood. Some hypotheses have been suggested. PEN could be a consequence of vascular lesions consecutively to infection with *Mycoplasma suis* and porcine circovirus type 2 (PCV-2), for example [2, 4, 8, 9]. The other hypotheses are triggered by the occurrence of ear traumas which may be linked to detrimental behaviours, like ear biting for example, together with infectious and non-infectious factors. Among non-infectious factors, there are management practices, poor housing, high humidity and temperature and mycotoxin contamination of feed [3, 10–12]. These conditions would favour infection of the ear tip by bacteria from the normal skin microbiota (as *Staphylococcus* spp.), or from the normal mouth microbiota of biters (as *Streptococcus* spp. and *Treponema* spp.) [3, 13]. Exfoliative staphylococcal toxins (mainly expressed by *Staphylococcus hyicus* and *Staphylococcus aureus*) may also be responsible for the damage of the epidermis of injured skin [3, 14, 15].

Bites are the main cause of behavioural trauma. Aggressive biting is common in the context of hierarchy formation and occurs mostly in the first hours after regrouping animals, and non-aggressive biting (also called oral manipulative behaviours) can occur at any time and largely results from the inability of pigs, in barren environment, to express natural behaviour such as rooting, chewing and foraging [16]. This urge to chew and root is then redirected towards any available materials in the environment, including pen mates and enrichment materials. However, there is a great variability among individuals in this motivation to bite. In a recent study, the occurrence of tail biting was associated with markers of oxidative stress and immune activation [17], but there are no data regarding the possible association with ear biting.

The aim of our study, carried out in a commercial post-weaning unit affected by PEN, was to improve the knowledge in the aetiology of PEN. First, we assessed whether the occurrence of PEN was associated with pig behaviours, which can traumatise the ear. Then, we studied the association of PEN severity with some blood health biomarkers, including haptoglobin as an indicator of inflammation, and hydroperoxides and blood antioxidant potential (BAP), as indicators of oxidative stress.

## Results

**Fig. 1:**
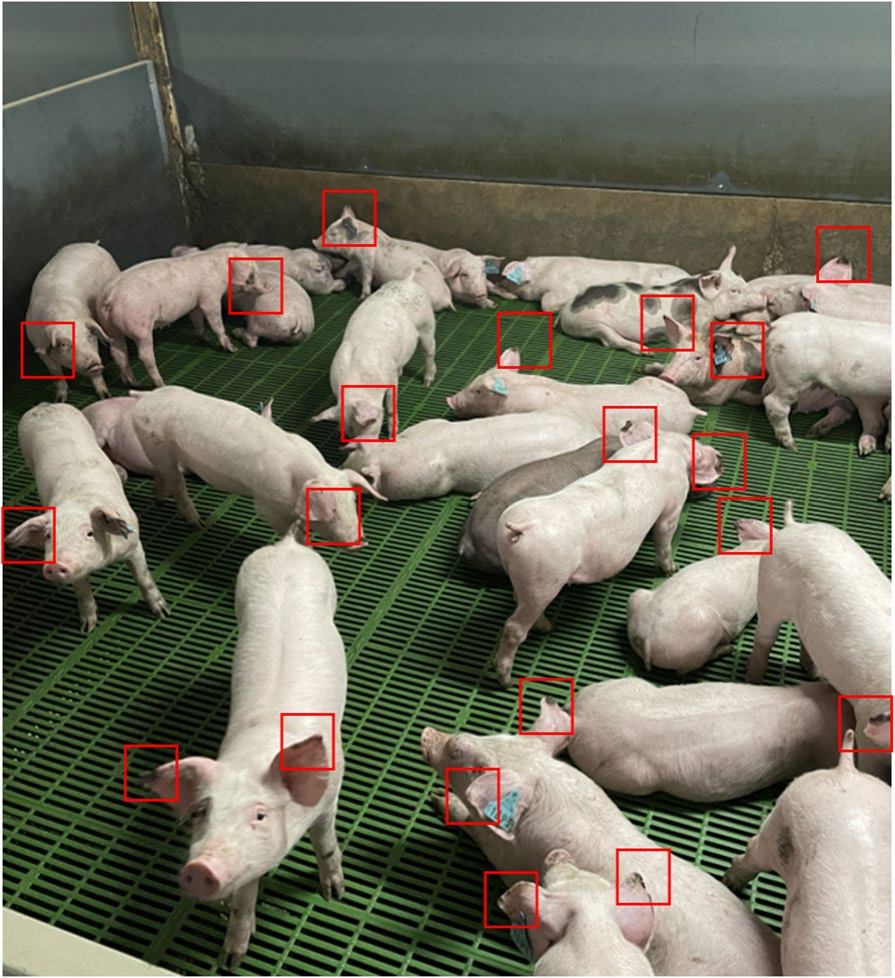
Example of pigs evaluated during the experiment. Red squares indicate ears with necrosis lesions

Temperature was 26.2 ± 0.5 °C and 27.2 ± 0.8 °C and RH was 56% ± 6 and 64% ± 4% in the first and the second batch respectively. The mean temperature and mean RH were in the same range during the early and the late post-weaning periods (Table 1). The percentage of pigs affected by PEN and the severity of the lesions on individually scored pigs are shown in Table 1. The proportions of pigs that could be scored for PEN on the photos were 53.4, 51.9 and 39.3% of pigs within a pen on average on D9, D30 and D50, respectively. An example of a part of a panoramic photo is presented in Fig. 1. Among blood sampled pigs, on which PEN severity was scored, 76.2 and 65.1% were affected by PEN on D30, and 64.3 and 42.9% were affected on D50, in the first and the second batch, respectively.

**Table 1:** Ambient temperature and percentage of relative humidity during periods D9-D30 and D31-D50 and PEN prevalence and severity at the end of these periods in both batches and both rooms of the experiment

		Period D9-D30		Period D31-D50	
		Room 1	Room 2	Room 1	Room 2
**Temperature (°C)**	Batch 1	26 ± 0.4	26.5 ± 0.4	26.0 ± 0.2	26.2 ± 1.1
	Batch 2	27.4 ± 0.6	27 ± 0.6	26.9 ± 0.8	26.5 ± 0.7
**Relative humidity (%)**	Batch 1	52.1 ± 4.4	52.9 ± 5.1	59.8 ± 3.4	61.1 ± 4.3
	Batch 2	63.7 ± 4.2	63.7 ± 3.9	63.8 ± 4.9	64.3 ± 5.1
**Pigs affected by PEN (%)**	Batch 1	67 ± 21	59 ± 19	76 ± 15	63 ± 27
	Batch 2	60 ± 27	62 ± 20	47 ± 19	58 ± 21
**PEN score**	Batch 1	0.95 ± 0.40	0.89 ± 0.56	1.11 ± 0.28	0.90 ± 0.42
	Batch 2	0.88 ± 0.48	0.95 ± 0.45	0.57 ± 0.52	0.87 ± 0.43

Data are presented as the mean ± standard deviations for each variable

Mean blood haptoglobin concentrations (mg/mL, ±SEM) decreased during the study: 2.7 ± 0.1 on D9, 1.6 ± 0.2 on D30 and 1.5 ± 0.2 on D50. Mean blood HPO concentrations (CARRU, ± SEM) decreased from 1201.1 ± 18.1 on D9 to 1193.5 ± 28.2 on D30 and then, increased up to 1326.2 ± 28.1 on D50. Mean blood BAP concentrations (μmol/L of eq vitC, ± SEM) increased from 2516.1 ± 18.4 on D9 to 2925.9 ± 26.9 on D30 and then decreased to 2845 ± 22.1 on D50. These kinetics in blood biomarkers were observed in both batches and both rooms during the experiment (Table 2).

**Table 2:** Blood concentrations of haptoglobin, hydroperoxides (HPO) and blood antioxidant capacity (BAP) per batch and per room during the experiment

		D9		D30		D50		SD^a^
Room		1	2	1	2	1	2	
Haptoglobin (mg/mL)	Batch 1	3.5	2.9	1.7	1.7	1.7	1.4	1.5
	Batch 2	2.2	2.2	1.5	1.4	1.5	1.6	1.8
HPO (CARRU)	Batch 1	1263	1186	1180	1186	1309	1288	259
	Batch 2	1154	1209	1198	1209	1321	1393	315
BAP (μmol/L of eq. vitamin C)	Batch 1	2447	2981	2853	2981	2726	2838	225
	Batch 2	2496	3018	2848	3018	2818	2997	301

^a^Data are presented as the means per room and per period, and for each parameter, only the maximal of the 6 standard deviations of the means (SD) is indicated

Piglets spent 53% of total observation time inactive, 16.9% engaged in social behaviours and 15% engaged in non-social exploratory behaviours. Locomotion and maintenance behaviours represented the last 15.1% of total observation time.

### Associations of behaviours with porcine ear necrosis at the pen level

For both periods (D9-D30 and D30-D50), the higher the time spent nosing pen mates during a period, the lower the proportion of pigs affected by PEN within the pen at the end of that period (*P <* 0.002) (Table 3). Regarding the expression of oral manipulation in the pen, during the second period (D30-D50), the higher the time spent manipulating orally pen mates, the higher the proportion of pigs affected by PEN on D50 (*P =* 0.03) (Table 3). No association was found between the time spent aggressing pen mates or orally manipulating the enrichment materials and the proportion of pigs affected by PEN.

**Table 3:** Proportion of time spent expressing specific behaviours during the two experimental periods and its association with the proportion of pigs affected by PEN at the end of the period (D30 for the period D9-D30 and D50 for the period D31-D50)

	Period D9-D30		Period D31-D50	
	%^A^	Β^B^	%^A^	Β^B^
Oral manipulation of pen mates	0.08 ± 0.06	0.7	0.20 ± 0.24	7.1*
Aggression of pen mates	0.09 ± 0.06	−1.8	0.06 ± 0.07	−0.05
Nosing pen mates	1.03 ± 0.36	−5.1*	0.71 ± 0.29	−3.8*
Oral exploration of the enrichment materials	0.18 ± 0.16	−2.2	0.17 ± 0.16	−0.2

^A^Proportion of scans, at the pen level, spent expressing specific behaviours (mean ± SEM)

^B^Slope coefficient of the model

* Indicates statistically significant coefficients (*P* < 0.05)

### Associations of blood biomarkers concentrations with porcine ear necrosis at the individual level

**Fig. 2:**
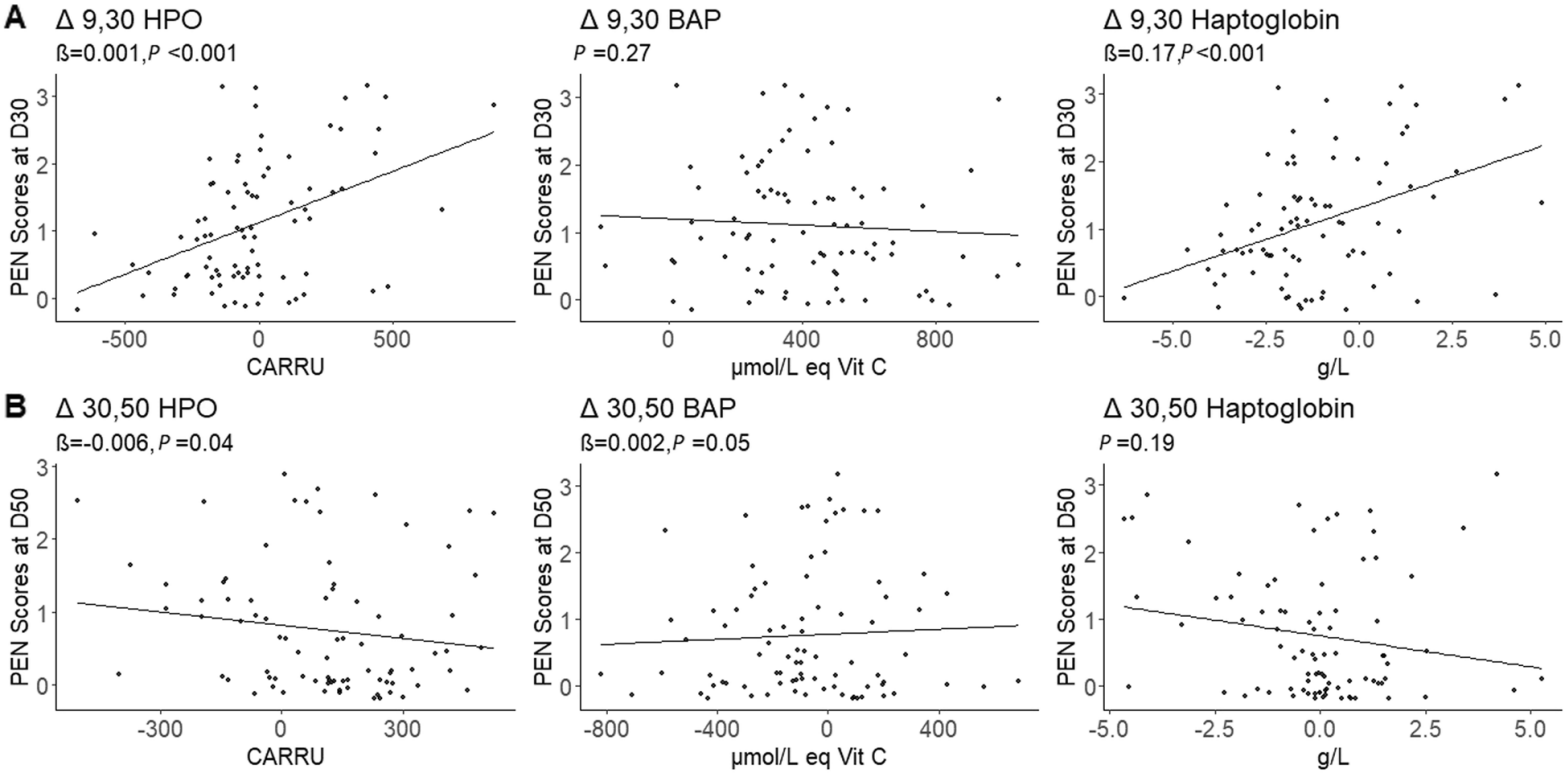
Mean porcine ear necrosis scores at blood sampled pig level on D30 (**A**) and D50 (**B**) depending on differences (Δ) in haptoglobin, hydroperoxides (HPO) and blood antioxidant potential (BAP) concentrations during the first (Δ9,30) (**A**) or during the second (Δ31,50) period (**B**)

During the trial, the mean PEN score of blood sampled pigs decreased significantly from 1.2 ± 0.8 on D30 to 0.6 ± 0.4 on D50 (*P < 0*.001). Correlations of blood biomarkers differences (Δ 9,30 and Δ 30,50) with PEN scores observed at the end of the same period are presented in Fig. 2. The higher the increases in HPO and in haptoglobin concentrations between D9 and D30, the higher PEN scores on D30 (*P <* 0.001 for both parameters). During the late post-weaning period (D30-D50), the higher the increase in BAP, the higher the PEN score on D50 (*P =* 0.05). In opposite, the higher the increase in HPO, the lower the PEN score on D50 (*P =* 0.04).

## Discussion

Porcine ear necrosis is a multifactorial condition in which infectious and non-infectious factors may play a significant role [2]. The aetiology and the pathogenesis of PEN are still largely unknown. Some non-infectious risk factors could be a source of discomfort and stress, and could be associated with a form of social instability. In this study, we investigated possible association between PEN syndrome and pigs’ social behaviours. Social interactions can be a source of stress and a cause of ear lesions, or a source of social cohesion and support, depending on the type of interaction. We did not observe any association between aggression and PEN, maybe because hierarchy, which is usually established during the first 48 hours after mixing at weaning [18], was already stabilized at the time of our observations. However, we observed that the higher the time spent nosing various parts of the body of pen mates, the lower the proportion of pigs affected by PEN at the pen level. In pigs, social nosing is a very frequent behaviour, which includes nose-to-body and nose-to-nose contacts [19]. It is considered a socio-positive behaviour, that is involved in social communication and recognition, and may thus help to maintain social cohesion within the group [20, 21]. In weanling pigs, a high level of social nosing may be an indicator of good social adaptation to weaning with less use of aggressive interactions [19]. Therefore, although we did not observe it because of the rareness of aggressive interactions in this study, groups with a better social cohesion may have presented less aggressive-biting behaviour. Whether this could lead to less ear injuries remains to be demonstrated. Tail and ear biting were previously suggested as a possible risk factor for PEN [3]. In the case of tail biting, it seems that the risk of both aggressive and non-aggressive tail biting decreases in groups of pigs with better social skills [16].

We also observed that high oral manipulations of pen mates in the pen increased the prevalence of PEN. Pigs have a strong need to root and forage which is not possible in barren and intensive pig housing. Oral manipulation of pen mates and the oral exploration of enrichment objects may have a similar motivational background [22]. This could explain why enriching the environment is a good way to reduce PEN [23]. Unfortunately, our recordings by scan sampling, initially designed for another purpose, were sub-optimal to detect rare and brief events, which might have generated a lack of accuracy and explain why we observed the association between PEN prevalence and oral manipulations only for the second observational period. Excessive oral manipulation of pen mates, including ear and tail biting, might have an impact on pig welfare because of the pain of bitten pigs but also because of the possibility of the spread of various pathogens. Overall, non-infectious factors probably have a major impact on the appearance of biting behaviours and ear lesions and consequently on PEN outbreaks but it requires further investigations. The temperature and the number of feeding places per pig in nursery, the flooring type both in the farrowing and the nursery units, the feed type in the growing unit and the overall hygiene policy were described as risk factors that could increase ear and tail biting during fattening [11]. Low availability of drinkers per pig [3] was also described as a significant risk factor. During our trial, the temperature and relative humidity in the pig housing, the feeder place and the availability of drinkers per pig fitted with the requirements in intensive pig production in Europe.

It seems reasonable to assume that ear lesions generate a local inflammatory state, which could perhaps reach a systemic level depending on the severity of the lesions. In this case, we can suppose that inflammatory biomarkers may reach detectable levels in pig blood. The concentrations of haptoglobin and HPO were probably greater on D9 than on D30 and D50 because of the proximity of weaning and transportation to the wean-to-finish unit. Indeed, the digestive disorders associated to weaning often peak between 10 and 20 days after weaning, and are associated with transient increases in plasma concentration of haptoglobin [24] and hydroperoxides [25]. Transportation and reallocation to a new housing is also a major stressful event that can lead to a rise in inflammatory markers [26]. However, as suboptimal health has been proposed to be a risk factor for tail biting [27], it also could be that indicators of inflammation could precede PEN development in a herd. In order to describe whether the onset of PEN could be related to elevated indicators of inflammation, three biomarkers were selected. During the early post-weaning period, the higher the increases in blood HPO and haptoglobin concentrations, the higher the PEN score at the end of the period, which appears coherent. An increase in haptoglobin during that period would indicate an increase in the inflammatory level of the pigs, and oxidative stress can also result from inflammatory situations [28]. However, during the late post-weaning period, PEN occurrence and severity was decreasing particularly in the second batch and all the more in one room without any treatment or change in management practices. This evolution during our experiment is difficult to explain. Comparing the severity and the evolution over time of PEN lesions with literature data is difficult as no consensus on the scoring system that should be used existed to date. During the late post-weaning period, the higher the increases in HPO and BAP, the lower and the higher the PEN score, respectively, which is difficult to explain. Moreover, it should be noticed that blood sampled animals were identified with ear tags on the day of the first blood sampling, which may have induced a bias in our PEN and inflammatory marker measures. Indeed, tagging generates an injury to the ear that other pigs of the pen did not have.

## Conclusion

High incidence of social nosing, which can be an indicator of good social cohesion in a group, was significantly associated with less frequent lesions of PEN in this study. In opposite, high incidence of oral manipulation of pen mates may increase the percentage of PEN-affected pigs. We failed to observe meaningful association of PEN with blood biomarkers of inflammation. Our results need to be confirmed in future investigations, but they suggest that PEN is a multifactorial condition which may have social causes among others.

## Methods

### Animals and housing

This study was performed between May and September 2021 in a wean-to-finish farm located in France. This farm was free from Porcine Reproductive and Respiratory Syndrome Virus. Two consecutive batches of weaned piglets (Large White x Landrace x Tai Zumu x Pietrain) were included. Piglets were born in a farrowing unit, their tails were docked and males were surgically castrated. They were weaned at 26 ± 1.5 days of age and moved to the wean-to-finish unit 4 to 5 days later (day 0 (D0, Table 4) of the trial, mean weight of 8.33 ± 1.79 kg). Piglets were vaccinated at inclusion (D0) against PCV-2 and *Mycoplasma hyopneumoniae*. In each batch, piglets were divided into two identical rooms, with 12 fully slatted (plastic gratings) pens per room and 34 ± 1.4 piglets per pen. Piglets were allocated to the pens with their littermates, and mixed with litters from sows of identical parity. In each pen, one steel feeder (4 cm per pig, dry feed *ab libitum*), two drinkers and two enrichment objects (one metal chain and one sub-optimal enrichment consisting in a wood block suspended to a metal chain) were installed. Temperature and relative humidity (RH) in rooms were continuously recorded using a data logger (Tinytag Plus 2 TGP-4500, Gemini Data Loggers, Chichester, United Kingdom). The feed came from an industrial feed mill and feed samples were collected at each delivery for mycotoxins contamination assessment using a liquid chromatography-electrospray ionization-tandem mass spectrometry technique (Labocéa, Ploufragan, France). All results for each mycotoxin and its derivates were below the detection limit of the method. No mycotoxin contamination was detected.

**Table 4:** Chronology of PEN lesions scoring, behaviour observation and blood sampling days

Day^A^	9	13	16	20	23	27	30	34	37	41	44	48	50
PEN observation^B^	+						+						+
PEN scoring							+						+
Blood sampling	+						+						+
Behavioural observation	+	+	+	+	+	+	+	+	+	+	+	+	

^A^Day 0: arrival to the wean-to-finish unit; ^B^Counts of the percentage of pigs affected by PEN as presence or absence

*PEN* porcine ear necrosis

### Prevalence and severity of porcine ear necrosis

PEN lesions were scored using a scoring method adapted from Pejsak et al. (2011): score 0, no lesion; score 1, ulcerative lesion and small crust on ear tip; score 2, mild necrotic lesions and crusts which could be sometimes suppurative and which affected less than 10% of the ear surface; score 3, severe necrotic lesions, crusts and suppurative lesions which affected more than 10% of the ear surface (Table 5).

**Table 5:**
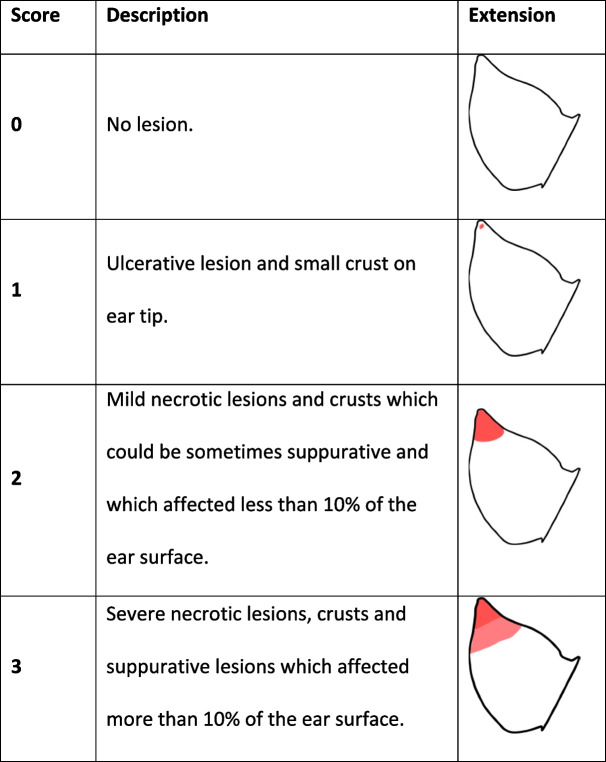
Ear lesions scoring in the experiment

The percentage of piglets affected by PEN was assessed at the pen level on D9, D30 and D50 by observation of panoramic photos of each pen (Fig. 1). Data were expressed as the number of pigs per pen with PEN lesions equal or higher than score 1 visible on at least one ear on the photo relatively to the total number of pigs with visible ears on the photo.

In each pen, two pigs were randomly selected (excluding pigs with omphalitis, arthritis and hernias) and ear tagged on D0. Those pigs were assigned an individual severity PEN score (mean of the scores of both ears) on D30 and D50.

### Blood sampling and analyses

For these two same pigs, on D9, D30 and D50, blood samples were collected by puncture of the jugular vein in 5-mL Vacutest® tubes containing heparin. Blood samples were stored on ice and centrifugated at room temperature at 3000 g for 15 minutes within 4 hours of collection. Plasma was stored at − 20 °C until analyses. Plasma concentration of haptoglobin (mg/mL) (Merlot et al., 2012), hydroperoxides (HPO) (Carratelli Unit, 1 CARRU = 0.08 mg H_2_O_2_/100 mL of sample) (Buchet et al., 2017) and blood antioxidant potential (BAP) (μmol/L of equivalent vitamin C) (Buchet et al., 2017) were analysed using commercial kits as previously described.

### Behavioural observations

On D0, two or three focal pigs (different from the ones selected for PEN severity scoring and blood sampling) were randomly selected per pen and identified with an extra ear tag, and colour paint sprayed on their back. After an 8-day period of habituation to the presence of the observer in the room, behaviours of the focal pigs were scored live. Behaviours were scored twice a week (Tuesday and Friday) for 6 weeks (from D9 to D48, Table 4), 1 hour in the morning (between 09.00 h and 11.00 h) and 1 hour in the afternoon (between 15.00 h and 17.00 h). The behaviour of each focal pig was recorded every 5 minutes using the scan sampling method, resulting in 24 scans per pig per day or 288 scans per pig in total. Of the 12 recorded activities (Table 6), three behaviours are presented here based on their potential role in the development of skin lesions: oral manipulation of pen mates, nosing of pen mates and aggression of pen mates. Moreover, the exploration and manipulation of enrichment materials was also included. Behavioural activities were expressed as proportions of total scans and were averaged per pen and per period (D9 to D30 and D30 to D50).

**Table 6:** Ethogram used in this experiment

Behaviours	Observation	Description
Inactive behaviours	Standing inactive	Standing on all four legs without performing any activity
	Lying inactive with eyes opened	Ventral or lateral recumbency with eyes open without performing any activity
	Lying inactive with eyes closed	Ventral or lateral recumbency with eyes closed and head resting on the floor
Social behaviours	Nosing pen mate while standing	Touching, gently rubbing, nibbling or licking the body of a pen mate with the snout while standing
	Aggressing pen mate	Head or shoulder knock, aggressively biting at any part of the body of a pen mate
	Manipulating orally pen mate	Biting, sucking, chewing or nibbling the tail, ear or any part of the body of a pen mates.
Non-social exploratory behaviours	Manipulating orally enrichment objects	Chewing, nosing, sniffing, touching or rooting on objects in the pen (chain or wooden toy) with the snout
	Pen surface exploration	Nosing, sniffing, touching or rooting on the floor or the pen fixtures with the snout, scraping the floor
Locomotion behaviours	Walking	Walking in the pen
Maintenance behaviours	Eating	Eating from the feeder or nosing, sniffing, touching, rooting the feeder with the snout
	Drinking	Drinking water from drinker or nosing, sniffing, touching, rooting the drinker with the snout
	Eliminating	Defecating or urinating

### Statistical analyses

Statistical analyses were conducted using R Studio (v 4.2.2, R Core Team, 2022). The normality (Shapiro-Wilk test) and variance homogeneity (Bartlett test) conditions were tested. The statistical significance threshold was set at *P ≤* 0.05, with 0.05 *< P ≤* 0.10 considered as a tendency. PEN scores per day were compared using Wilcoxon tests. Then, two periods were defined: the early (D9–30) and late (D30–50) post-weaning periods. Each pen was characterized by a percentage of pigs affected by PEN, a mean PEN score (mean of individual PEN score of blood sampled pigs within a pen), mean behavioural expressions (mean of individual proportions of behaviour expression of focal pigs within a pen), and mean blood biomarker concentrations (mean of the two blood sampled pigs per pen). For each behaviour, pens were divided into two categories to compare pens in which the behaviour was over expressed with the other pens: one group in which the time spent exhibiting the behaviour was in quartile 1 to quartile 3, and one group in which the time spent exhibiting the behaviour was in quartile 4. A generalized linear mixed model, with pen and batch as random effects, and behavioural categories as fixed effects were applied to analyse the percentage of pigs affected by PEN. Mixed linear models, with the behavioural category as fixed effect, and pen and batch as random effects, were used to assess the effect of high level of behavioural expressions on PEN scores (severity of PEN). Differences (Δ) in haptoglobin, HPO and BAP concentrations between sampling times were calculated per period (Δ9,30 = D30-D9 and Δ30,50 = D50-D30). At the pen level, the percentage of pigs affected by PEN was analysed separately at each period with a generalized linear mixed model, with pen and batch as random effects, and blood biomarker variations (haptoglobin, HPO and BAP) as covariates. At the individual level, PEN scores at the end of each period were analysed with mixed linear models with pen as random effect and Δ9,30 (for D30) or Δ30,50 (for D50) blood parameter variation as covariates. Finally, Pearson correlation coefficients were calculated when the assumption of normality was verified; otherwise Spearman correlation coefficients were calculated.

## Data Availability

The datasets used and/or analysed during the current study are available from the corresponding author on reasonable request.

## Abbreviations

BAP: Blood Antioxidant Potential
CARRU: Carratelli Unit
D: Day
HPO: Hydroperoxides
PEN: Porcine Ear Necrosis
RH: Relative Humidity
SEM: Standard Error to the Mean

## References

[1] Kureljušić B, Savić B, Milićević V, Jezdimirović N, Radanović O, Žutić J (2021). Investigation of possible aetiological/triggering factors in porcine ear necrosis syndrome at a farrow-to-feeder pig system. Acta Vet Hung.

[2] Malik M, Chiers K, Boyen F, Croubels S, Maes D (2021). Porcine ear necrosis. Vet J.

[3] Park J, Friendship RM, Poljak Z, DeLay J, Slavic D, Dewey CE (2013). An investigation of ear necrosis in pigs. Can Vet J.

[4] Pejsak Z, Markowska-Daniel I, Pomorska-Mól M, Porowski M, Kołacz R (2011). Ear necrosis reduction in pigs after vaccination against PCV2. Res Vet Sci.

[5] Petersen HH, Nielsen EO, Hassing AG, Ersbøll AK, Nielsen JP (2008). Prevalence of clinical signs of disease in Danish finisher pigs. Vet Rec.

[6] Malik M, Schoos A, Chantziaras I, Donkers D, Croubels S, Doupovec B (2021). Porcine ear necrosis in weaned piglets: prevalence and impact on daily weight gain. Porcine Health Manag.

[7] Malik M, Chiers K, Theuns S, Vereecke N, Chantziaras I, Croubels S (2023). Porcine ear necrosis: characterization of lesions and associated pathogens. Vet Res.

[8] Olson LD (1981). Gross and microscopic lesions of middle and inner ear infections in swine. Am J Vet Res.

[9] Truszczyński M, Pejsak Z (2009). Mycoplasma suis and porcine eperythrozoonosis, including achievements of the last years. Medycyna Weterynaryjna.

[10] Diana A, Boyle LA, García Manzanilla E, Leonard FC, Calderón Díaz JA (2019). Ear, tail and skin lesions vary according to different production flows in a farrow-to-finish pig farm. Porcine Health Manag.

[11] Smulders D, Hautekiet V, Verbeke G, Geers R (2008). Tail and ear biting lesions in pigs: an epidemiological study. Anim Welf.

[12] Weissenbacher-Lang C, Voglmayr T, Waxenecker F, Hofstetter U, Weissenböck H, Hoelzle K (2012). Porcine ear necrosis syndrome: a preliminary investigation of putative infectious agents in piglets and mycotoxins in feed. Vet J.

[13] Pringle M, Backhans A, Otman F, Sjölund M, Fellström C (2009). Isolation of spirochetes of genus Treponema from pigs with ear necrosis. Vet Microbiol.

[14] Mirt D (1999). Lesions of so-called flank biting and necrotic ear syndrome in pigs. Vet Rec.

[15] Richardson JA, Morter RL, Rebar AH, Olander HJ (1984). Lesions of porcine necrotic ear syndrome. Vet Pathol.

[16] Prunier A, Averos X, Dimitrov I, Edwards SA, Hillmann E, Holinger M (2020). Review: early life predisposing factors for biting in pigs. Animal.

[17] Sánchez J, Matas M, Ibáñez-López FJ, Hernández I, Sotillo J, Gutiérrez AM. The connection between stress and immune status in pigs: a first salivary analytical panel for disease differentiation. Front Vet Sci. 2022; [cited 2023 Jul 4];9. Available from: https://www.frontiersin.org/articles/10.3389/fvets.2022.881435 10.3389/fvets.2022.881435 PMC9244398 35782547

[18] Meese GB, Ewbank R (1973). The establishment and nature of the dominance hierarchy in the domesticated pig. Anim Behav.

[19] Clouard C, Resmond R, Vesque-Annear H, Prunier A, Merlot E (2023). Pre-weaning social behaviours and peripheral serotonin levels are associated with behavioural and physiological responses to weaning and social mixing in pigs. Appl Anim Behav Sci.

[20] Camerlink I, Turner SP, Ursinus WW, Reimert I, Bolhuis JE (2014). Aggression and affiliation during social conflict in pigs. PLoS One.

[21] Camerlink I, Turner SP (2013). The pig’s nose and its role in dominance relationships and harmful behaviour. Appl Anim Behav Sci.

[22] Camerlink I, Ursinus WW, Bijma P, Kemp B, Bolhuis JE (2015). Indirect genetic effects for growth rate in domestic pigs alter aggressive and manipulative biting behaviour. Behav Genet.

[23] Schmitt O, Poidevin A, O’Driscoll K (2020). Does diversity matter? Behavioural differences between piglets given diverse or similar forms of enrichment pre-weaning. Animals.

[24] Gilbert H, Ruesche J, Muller N, Billon Y, Begos V, Montagne L (2019). Responses to weaning in two pig lines divergently selected for residual feed intake depending on diet1. J Anim Sci.

[25] Buchet A, Belloc C, Leblanc-Maridor M, Merlot E (2017). Effects of age and weaning conditions on blood indicators of oxidative status in pigs. PLoS One.

[26] Averós X, Herranz A, Sánchez R, Gosálvez LF (2009). Effect of the duration of commercial journeys between rearing farms and growing–finishing farms on the physiological stress response of weaned piglets. Livest Sci.

[27] Valros A, Heinonen M (2015). Save the pig tail. Porcine Health Manag.

[28] Durand D, Collin A, Merlot E, Baéza E, Guilloteau LA, Le Floc’h N (2022). Review: implication of redox imbalance in animal health and performance at critical periods, insights from different farm species. Animal.

